# Tolerance of three grain legume species to transient waterlogging

**DOI:** 10.1093/aobpla/plv040

**Published:** 2015-04-22

**Authors:** Al Imran Malik, Tadhamin Iskander Ailewe, William Erskine

**Affiliations:** 1 Centre for Plant Genetics and Breeding (PGB) and The Institute of Agriculture, The University of Western Australia, 35 Stirling Highway, Crawley, WA 6009, Australia; 2 School of Plant Biology, The University of Western Australia, 35 Stirling Highway, Crawley, WA 6009, Australia

**Keywords:** Chlorophyll, intra-species variation, legume, porosity, waterlogging

## Abstract

Waterlogging reduces the yield of food crops. Tolerance to waterlogging could vary between and within species. This study aimed to quantify tolerance to soil waterlogging in two divergent genotypes of pea (*Pisum sativum*), two of lentil (*Lens culinaris*) and a grasspea (*Lathyrus sativus*) control at germination and during vegetative growth. Following germination, seeds were grown for 14 days in soil waterlogged with the water table 10 mm below the surface, and then by draining the pots and allowing to recover for 21 days—to be compared with 35 days of continuous waterlogging. In both pea and lentil, the pair of genotypes contrasted widely with large-seeded pea genotype Kaspa and lentil genotype Nugget showing higher (2-fold) root porosity and less effect on shoot nitrogen content under waterlogging than the other genotypes (NPE and ATC). During recovery, the same two genotypes—Kaspa pea and Nugget lentil—also recovered better than their smaller-seeded species pairs. Soil waterlogging at 10 mm depth had no significant effect on shoot and root dry mass after 14 days. Root penetration into waterlogged soil was restricted to ∼100 mm depth and its distribution altered for pea and lentil genotypes but not for grasspea. Within the small sample studied, we demonstrated a significant genetic variation in both pea and lentil in tolerance to waterlogging after germination and subsequent recovery for the first time. Screening of additional pea and lentil germplasm for waterlogging conditions is clearly warranted.

## Abstract (precis)

Crop production can be limited by soil waterlogging. Tolerance to waterlogging can vary between and within species. This study quantified tolerance to soil waterlogging in two divergent genotypes of pea (*Pisum sativum*), two of lentil (*Lens culinaris*) and a grasspea (*Lathyrus sativus)* control at germination and during vegetative growth. Soil waterlogging at 10 mm depth had no significant effect on shoot and root dry mass after 14 days. Significant genetic variation in both pea and lentil in tolerance to waterlogging after germination and subsequent recovery was evident. Screening of additional pea and lentil germplasm for waterlogging conditions is clearly warranted.

## Introduction

Soil waterlogging is a common abiotic stress that impacts on crop production (Jackson and Colmer 2005). Seeds of some species can withstand waterlogged conditions (i.e. prolonged soaking) more than others (Crawford 1977). The effects of hypoxia and/or anoxia during vegetative growth of dryland crops are well documented; shoot and root growth decrease and nutrient uptake becomes inhibited (pea, Cannell *et al*. 1979; lupin, Davies *et al*. 2000; wheat, Malik *et al*. 2002; barley, Pang *et al*. 2004; chickpea, Palta *et al*. 2010). Photosynthesis is also severely inhibited in waterlogging-sensitive plants resulting in reduced dry matter accumulation (wheat, Malik *et al*. 2001; barley, Pang *et al*. 2004). Inhibition of nitrogen nutrition reduces chlorophyll concentration of leaves resulting in premature leaf senescence (wheat, Malik *et al*. 2002; soya bean, Youn *et al*. 2008). Increased porosity through the development of aerenchyma tissue in root systems enhances the tolerance to waterlogging by facilitating oxygen movement via diffusion from shoots to roots (Colmer 2003). However, the response of a crop to waterlogging depends on the timing (developmental stage of the plant when stress was imposed), duration (number of days) and genotypic variation in waterlogging tolerance (Setter and Waters 2003).

Food legumes, a major dietary protein source (Erskine 2009), are grown on 77 million hectare worldwide [Food and Agriculture Organization (FAO) 2013] and often affected by waterlogging. Legume crops such as lupin (*Lupinus angustifolius*), chickpea (*Cicer arietinum*), lentil (*Lens culinaris* subsp. *culinaris*) and field pea (*Pisum sativum*) are susceptible to waterlogging at vegetative stages (Siddique *et al*. 1993; Siddique and Sykes 1997; Yu and Rengel 1999; Palta *et al*. 2010). In a detailed comparison of the differing responses of food legumes to waterlogging during vegetative growth, Solaiman *et al*. (2007) found faba bean (*Vicia faba*) as the most tolerant and field pea the least; with yellow lupin (*Lupinus luteus*), grasspea (*Lathyrus sativus*), narrow-leafed lupin, chickpea and lentil as intermediate (Solaiman *et al*. 2007). Within-species genetic variation in waterlogging tolerance is present in lupin (Davies *et al*. 2000), faba bean (Solaiman *et al*. 2007), soya bean (Henshaw *et al*. 2007), lotus (Real *et al*. 2008) and chickpea (Palta *et al*. 2010).

Waterlogged soil restricts food legume establishment in rice-based cropping systems in the Eastern Gangetic Plains of Bangladesh, Eastern India and Nepal (Ali *et al*. 2009). In such systems, pea, lentil, chickpea, grasspea and soya bean (*Glycine max*) are sown on residual soil moisture after paddy rice (Awadhwal *et al*. 2001). Relay sowing, the practice of broadcasting seed into a standing rice crop prior to harvest, predominates in Nepal for lentil and in Bangladesh for grasspea, and relay sown pea is also found (A. I. Malik, pers. obs.). Malik *et al.* (2015) recently found that the substitution of relay sown lentil for fallow was a useful option to intensify cropping in the Eastern Gangetic Plain. At the time of relay sowing, excess soil moisture and puddles are common features in puddled soil and such crops face transient soil waterlogging from an early developmental stage. Crawford (1977), Jackson (1979) and Sarlistyaningsih *et al*. (1995) demonstrated variation in germination of pea, bean and lupin seeds under different durations of waterlogging or anoxic conditions. Poor germination in waterlogged soil is a major impeding factor for crop establishment (Ramakrishna *et al*. 2000). Setter and Waters (2003) emphasized the need for future research on waterlogging tolerance during germination. Knowledge on waterlogging tolerance from germination to crop establishment in lentil and pea is scant. This study aimed to assess waterlogging tolerance in contrasting pairs (seed size and origins—Table 1) of pea and lentil genotypes—compared with a grasspea, considered waterlogging tolerant (Purseglove 1968), control—in the period after germination to vegetative growth and during subsequent recovery.

**Table 1.** Names, country of origin and 100 seed weight of five legume genotypes.

Crop	Name	Origin	100 seed weight (g)
Pea	Kaspa	Australia	27.3 ± 1.4
	NPE 1191.515	Pakistan	7.2 ± 0.3
Lentil	Nugget	Australia	4.1 ± 0.5
	ATC 70856	Bangladesh	1.8 ± 0.6
Grasspea	Ceora	Australia	11.4 ± 1.1

## Methods

### Experimental design

The experimental design was factorial with treatments of genotypes (5) (Table 1) × waterlogging (3) in a completely randomized block design with four replications. A pot was the experimental unit. Genotype pairs (pea and lentil) were selected for their contrasting seed size and origins (Table 1).

Genotypes were sown in pots either free-drained (control) or waterlogged to 10 mm below the soil surface at the start of the experiment. This water table depth best represents relay sowing conditions in the field (Ali 2011). Fourteen days after sowing (DAS), waterlogged pots were either continued as waterlogged or allowed to drain. This gave the three treatments: (i) drained control, (ii) waterlogged (i.e. continuously waterlogged for 35 days) and (iii) recovery (14 day of waterlogging followed by 21 days recovery).

After 14 DAS, 4 waterlogged and 4 control pots were harvested (H1); and 4 pots were allowed to drain from each genotype. The pots were randomly re-positioned every 7 days to minimize glasshouse positional effects.

The experiment was conducted during the winter (July–August) of 2012 in the glasshouse of the University of Western Australia Plant Growth Facility. For the period of the experiment, minimum and maximum temperatures during the day were 16.9 and 23.9 °C, respectively; and photoperiod 10.5 h, light intensity 1000 µmol m^−2^ s^−1^.

#### Plant culture

Seeds of two pea, two lentil and a grass pea genotype(s) (Table 1) were surface sterilized with 1 % commercial bleach (active ingredients NaOCl 40 mg L^−1^) for 1 min, washed 3–5 times with deionized water and placed on moist filter paper (Whatman no. 1) in 90 mm Petri dishes in a dark cabinet at room temperature overnight to imbibe. Seeds of grasspea were imbibed 3 days before the other genotypes (grasspea took longer to imbibe in a preliminary trial).

Four imbibed seeds were sown in each pot for lentil and grasspea and two seeds for pea genotypes. Seeding rate in the pots was decided according to reported field rates [i.e. 100–120 seeds m^−2^ for lentil and 45–55 seeds m^−2^ for pea (White *et al*. 2005)]. Seeds were placed on the surface of free-draining plastic pots (height 140 mm, diameter 120 mm) and each pot was placed in a closed-bottom plastic pot (height 180 mm, diameter 200 mm). Waterlogged treatment was imposed by filling the closed-bottom pots with DI-H_2_O up to 10 mm below the soil surface of free-draining pots. Water table was maintained by adding water every day. Control pots were weighed to 80 % of the field capacity at the start and retained at that weight by adding water every second day. Pots contained gravel at the bottom and 1.2 kg of mixed washed river sand and potting mix (1 : 1) (pH = 6.3 and EC (1 : 5 weight/volume in water extract) = 0.197 dS m^−1^). The potting mix contained 5 : 2 : 3 composted pine bark, coco peat and brown river sand. Rhizobium was not added to substrate. While filling the pots, platinum (Pt) electrodes were inserted in the substrate in 15 pots at a depth of 100 mm for redox measurement with three treatments for each genotype. Potting mix and river sand contained $\text{N}{{\text{H}}_{4}}^{+}$ 0.004 and 0.018, $\text{N}{{\text{O}}_{3}}^{-}$ 0.062 and 0.04, K^+^ 0.0005 and 0.0009 μmol kg^−1^ substrate, respectively.

The following nutrients were applied once 15 DAS which allow plants to establish on seed reserve prior to fertilization. Nutrients (μmol kg^−1^ substrate): KNO_3_ 0.15, Ca(NO_3_)_2_ · 4H_2_O 0.375, KH_2_PO_4_ 0.191, MgSO_4_ · 7H_2_O 0.025, KCl 0.0030, H_3_BO_3_ 0.0012, MnSO_4_ · H_2_O 0.0002, ZnSO_4_ · 7H_2_O 0.0005, CuSO_4_ · 5H_2_O 0.00008, Na_2_MoO_4_ · 2H_2_O 0.00008 and NiSO_4_ · 6H_2_O 0.00025.

### Measurements

#### Redox

Redox potential was measured daily in 15 pots (i.e. 6 waterlogged, 6 recovery and 3 control) with Pt electrodes and a silver/silver chloride reference electrode attached to a millivolt-meter. The reading was corrected as described by Patrick *et al*. (1996).

#### Growth

Harvested plants were divided into shoots and roots. All plant parts were dried for 72 h at 59 °C. Plant biomass at H1 and final harvest was measured by weighing the dry mass of the shoots and roots. The root lengths of main axis and the longest lateral roots were measured with a ruler at harvest.

#### Porosity

Porosity (% gas space per volume) of main and lateral roots was determined at in the final harvest following the principle described by Raskin (1983) and as modified by Thomson *et al*. (1990). Briefly, roots were lined up and cut into 50 mm segments on a tray containing water to avoid root drying. Roots were weighed submerged and then vacuum infiltration was carried out by subjecting the submerged tissue to low pressure with a vacuum pump three times to ensure the exit of all air from the roots. The fresh mass of the roots in the air was recorded and corrected using the equation of Thomson *et al*. (1990).

#### Nitrogen, chlorophyll concentration and chlorophyll fluorescence

Oven-dried pulverized samples were analysed for total nitrogen (N) in shoots using an auto analyser (Elementer, Model: Vario Macro, Hanau, Germany) against ethylenediaminetetraacetic acid and rice flour as standards. Total N was calculated on shoot dry mass basis. Relative changes in chlorophyll concentration were assessed thrice during the experiment at 21, 27 and 34 DAS on the youngest fully-expanded leaves of all plants in each pot with a hand-held chlorophyll meter (Minolta SPAD 502, Osaka, Japan).

Chlorophyll fluorescence was measured at 34 DAS on the youngest fully-expanded leaves of 1 plant in each pot after keeping the leaves in the dark for 30 min prior to measurement by using plant efficiency analyser (PEA) (Hansatech Instrument Ltd, UK). Measurements were taken at 50 % light intensity and the exposure time was set at 5 s. To obtain optimum light saturation point, a series of measurements taken in a preliminary experiment to ensure photosynthetic apparatus response to light and the accuracy of measurement for fluorescence. The *F*_0_ (minimal fluorescence), *F*_m_ (maximal fluorescence), *F*_v_ (variable fluorescence) and *F*_v_/*F*_m_ were recorded.

### Statistical analysis

Two-way analyses of variance (ANOVAs) were performed using GenStat 14th edition (VSN International) to determine the effects of plant genotype (2 pea, 2 lentil and 1 grass pea), waterlogging (well drained, waterlogged for 14 days, waterlogged for 35 days) and their interaction on the following response variables: main root length, main root porosity, shoot chlorophyll and nitrogen concentration, shoot and root mass. If main effects or interactions were significant, we then proceeded with multiple comparison tests to compare differences among means using Tukey's test. Separate analyses were performed on data collected by Day 14 and on data collected after 35 days of treatment.

## Results

### Redox potential

Redox potential was 365 ± 23 mV in the drained control pots and 200 ± 4 mV in the waterlogged pots at the start of the experiment. After 14 days the redox potential had declined to 173 ± 40 mV and it was 70 ± 10 mV by 35 days in waterlogged pots at the end of the experiment. After draining waterlogged pots on 14 DAS, the redox potential increased to 248 ± 55 mV after 2 days and to 403 ± 21 mV after 7 days of recovery. The redox potential of control pots remained close to 400 mV throughout the experimental period (data not shown).

### Root growth

**Figure 1.**
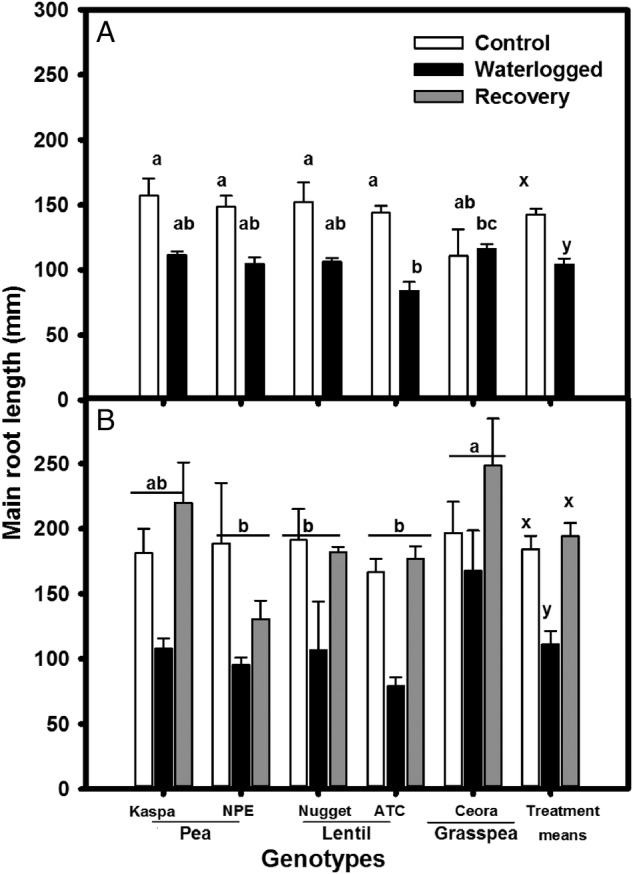
Main root length (mm) of legume genotypes (A) after 14 days and (B) after 35 days of waterlogging. Treatments were drained control (white bars), continuously waterlogged (black bars), 14 days waterlogged and subsequent 21 days of recovery (grey bars). Values are the means of four replicates standard errors. Overall treatment (+SE) means are shown grouped on the right. Means associated with different letters are significantly different (*P* < 0.05) by Tukey's test.

Roots of plants grown in drained soil reached the base of pots after 14 days of treatment for all genotypes. Overall root length was significantly (*P* < 0.001, Table 2) shorter in plants grown in waterlogged soil for 14 days than in the drained control in all pea and lentil genotypes (Fig. 1A). However, root length in grasspea was unaffected by waterlogging (Fig. 1A).

**Table 2.** Degrees of freedom (df), *F* values and probabilities of two-way ANOVA. ^1^Values in parentheses indicate number of missing plots.

Character	Source of variation	Genotype (G)	Treatment (T)	G × T	Residual
		Waterlogging 14 days			
Shoot mass	df	4	1	4	25 (5)^1^
	*F* value	9.11	0.05	1.24	
	Probability	<0.001	0.825	0.318	
Root mass	*F* value	9.31	2.82	0.86	
	Probability	<0.001	0.105	0.502	
Main root length	*F* value	1.31	29.28	3.12	
	Probability	0.295	<0.001	0.033	
Shoot nitrogen	df	4	1	4	22 (8)^1^
	*F* value	18.31	21.60	0.49	
	Probability	<0.001	0.002	0.746	
		Waterlogging 35 days			
Shoot mass	df	4	2	8	42 (3)^1^
	*F* value	88.01	0.56	0.30	
	Probability	<0.001	0.574	0.961	
Root mass	*F* value	21.85	1.13	0.26	
	Probability	<0.001	0.331	0.976	
Main root length	*F* value	4.30	19.54	1.09	
	Probability	0.005	<0.001	0.388	
Shoot nitrogen	*F* value	2.55	30.93	3.17	
	Probability	0.053	<0.001	0.007	
Chlorophyll concentration	*F* value	31.63	341.93	29.01	
	Probability	<0.001	<0.001	<0.001	
Chlorophyll fluorescence	*F* value	2.09	4.59	1.60	
	Probability	0.100	0.016	0.153	
Root porosity	df	4	2	8	30 (15)^1^
	*F* value	8.59	6.07	0.99	
	Probability	<0.001	0.006	0.463

Again with the exception of grasspea, the extended waterlogging treatment stopped main root extension in all genotypes when the duration of waterlogging was prolonged from 14 to 35 days (Fig. 1A and B). On those pots, where the waterlogging stress was ceased after 14 days, main roots continued to elongate and reach the length of roots on continuously drained pots in all genotype except pea—NPE (Fig. 1B).

As with root length, root dry mass differed significantly (*P* < 0.001, Table 2) among legume genotypes when grown in drained soil; however, there was no effect of waterlogging on root dry mass (Table 3).

**Table 3.** Root mass of five legume genotypes after 14 and 35 days of treatment in control, fully-drained soil; waterlogged, water table 10 mm below soil surface; recovery, after 14 days of waterlogging pots were allowed to drain. Means associated with different letters are significantly different (*P* < 0.05) by Tukey's test. Values are the means of four replicates.

Crop	Name	Root mass (mg plant^−1^) 14 days			Root mass (mg plant^−1^) 35 days			
		Control	Waterlogged	Mean	Control	Waterlogged	Recovery	Mean
Pea	Kaspa	114 ± 22	91 ± 20	102.5 ac	359 ± 12	391 ± 30	348 ± 30	332.5 a
	NPE	42 ± 10	40 ± 9	40.9 ab	432 ± 14	384 ± 110	433 ± 90	416.3 a
Lentil	Nugget	55 ± 10	38 ± 8	46.6 ab	177 ± 23	133 ± 11	169 ± 32	159.6 bc
	ATC	20 ± 7	27 ± 8	23.3 c	97 ± 21	46 ± 1	84 ± 3	75.6 c
Grasspea	Ceora	90 ± 20	48 ± 4	69.6 ab	151 ± 110	192 ± 20	244 ± 24	195.8 b

### Porosity of main and lateral roots

**Figure 2.**
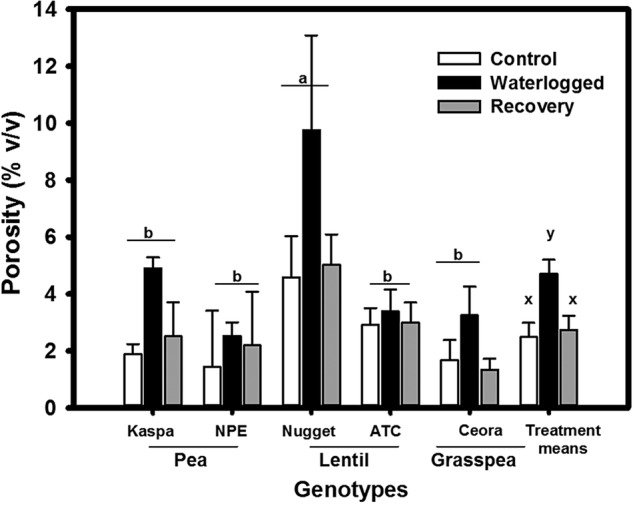
Effect of waterlogging treatment on root systems of legume genotypes main root porosity (% gas per unit volume after 35 days of growth. Treatments were drained control (white bars), continuously waterlogged (black bars), 14 days waterlogged and subsequent 21 days of recovery (grey bars). Values are the means of four replicates standard errors. Overall treatment (+SE) means are shown grouped on the right. Means associated with different letters are significantly different (*P* < 0.05) by Tukey's test.

In drained soil, main root porosity was highest (i.e. 4.6 %) for lentil—Nugget; and this value was ∼2-fold higher than any other legume genotype in the study. Thirty-five days of waterlogging significantly (*P* > 0.001, Table 2) increased main root porosity in all legume genotypes (Fig. 2). Porosity increased for Kaspa, Nugget and grasspea by 2-fold, NPE by 1.1-fold and ATC by 1.4-fold in plants grown in waterlogged soil compared with the drained control.

Main root (i.e. tap root) porosity of plants that were allowed to drain after 14 days of waterlogging returned towards the control value in all genotypes (Fig. 2A) due to resumed growth after drainage of the pots. Lateral root porosity demonstrated a similar pattern to main roots when grown in drained and in waterlogged soil as well as at recovery (data not shown). Waterlogging increased (both main and lateral) porosity over the control due to the formation of aerenchyma (visual observation under microscope—data not shown).

### Shoot growth and chlorophyll concentration

**Figure 3.**
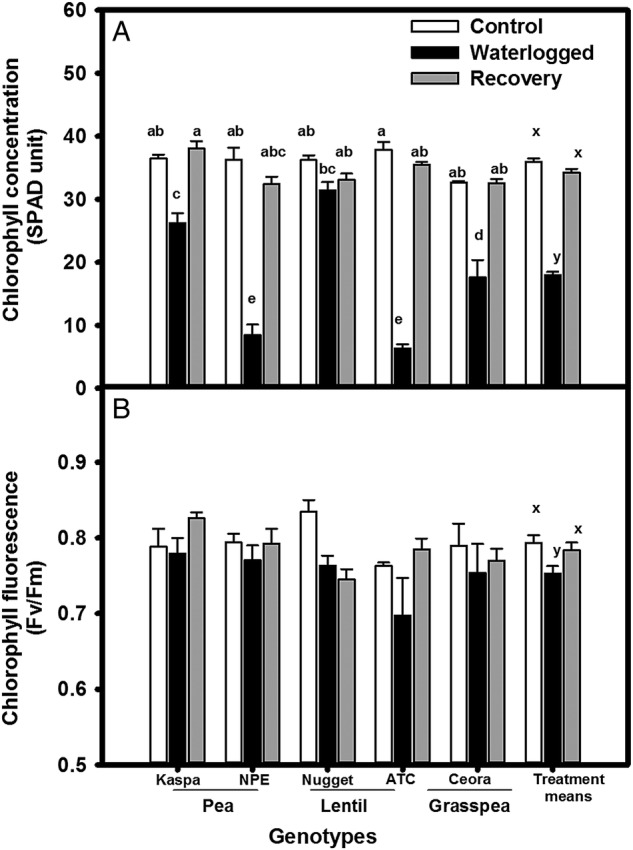
Effect of waterlogging treatment on youngest fully-expanded leaves. (A) Chlorophyll concentration (SPAD unit) and (B) chlorophyll fluorescence (*F*_v_/*F*_m_) of legume genotypes after 35 days of growth. Treatments were drained control (white bars), continuously waterlogged (black bars), 14 days waterlogged and subsequent 21 days of recovery (grey bars). Values are the means of four replicates standard errors. Overall treatment (+SE) means are shown grouped on the right. Means associated with different letters are significantly different (*P* < 0.05) by Tukey's test.

Shoot dry mass differed significantly among legume genotypes grown in drained soil. Waterlogging had no effect on shoot dry mass compared with the drained control (Table 4). However, waterlogging reduced leaf chlorophyll concentration significantly in all genotypes when compared with the drained control (*P* < 0.001, Table 2) (Fig. 3A). At the end of the waterlogging treatment period (35 DAS), there was a significant interaction (*P* < 0.001, Table 2) of treatment × within-species variation, indicating that chlorophyll concentration in small-seeded pea (NPE) and lentil (ATC) were markedly reduced compared with the larger-seeded pea Kaspa and lentil Nugget. After only 7 days of recovery, chlorophyll concentration started to recover (data not shown) and recovered to the control value by the end of the experiment (Fig. 3A).

**Table 4.** Shoot mass of five legume genotypes after 14 and 35 days of growth in control, fully-drained soil; waterlogged, water table 10 mm below soil surface; recovery, after 14 days of waterlogging pots were allowed to drain. Means associated with different letters are significantly different (*P* < 0.05) by Tukey's test. Values are the means of four replicates.

Crop	Name	Shoot mass (mg plant^−1^) 14 days			Shoot mass (mg plant^−1^) 35 days			
		Control	Waterlogged	Mean	Control	Waterlogged	Recovery	Mean
Pea	Kaspa	84 ± 21	59 ± 20	71.3 a	1680 ± 140	1406 ± 140	1475 ± 250	1496 a
	NPE	42 ± 4	60 ± 20	50.8 ab	1157 ± 110	1014 ± 110	1060 ± 200	1077 b
Lentil	Nugget	21 ± 4	23 ± 2	21.7 b	293 ± 40	232 ± 52	274 ± 23	266 c
	ATC	25 ± 10	32 ± 11	28.6 b	194 ± 31	155 ± 24	202 ± 33	184 c
Grasspea	Ceora	20 ± 3	26 ± 2	21.9 b	341 ± 110	427 ± 32	429 ± 53	399 c

Chlorophyll fluorescence (maximum quantum efficiency *F*_v_/*F*_m_) on the youngest fully developed leaves was reduced by waterlogging in all legume genotypes compared with drained control (*P* < 0.05, Table 2) (Fig. 3B). The reduction was more pronounced in lentil than in pea. The chlorophyll fluorescence of the youngest fully-expanded leaf of plants previously waterlogged recovered to the control value for all legumes except for lentil Nugget (Fig. 3B).

### Nitrogen concentration

**Figure 4.**
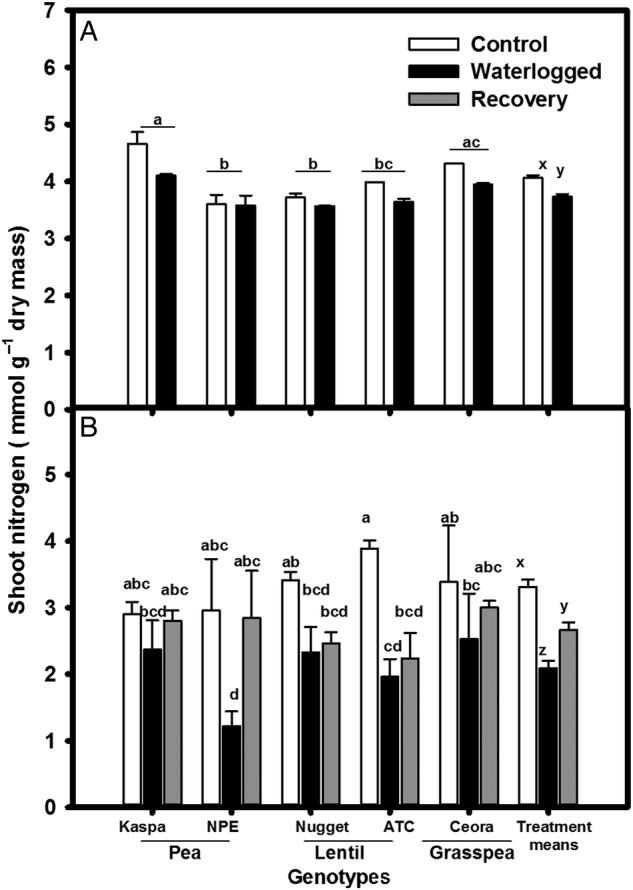
Effect of waterlogging treatment on shoot nitrogen (mmol g^−1^ dry mass) of legume genotypes (A) after 14 days and (B) after 35 days of growth. Treatments were drained control (white bars), continuously waterlogged (black bars), 14 days waterlogged and subsequent 21 days of recovery (grey bars). Values are the means of four replicates standard errors. Overall treatment (+SE) means are shown grouped on the right. Means associated with different letters are significantly different (*P* < 0.05) by Tukey's test.

Total shoot nitrogen concentration decreased over time in all plants grown in drained conditions (Fig. 4A and B). Fourteen days of waterlogging significantly (*P* < 0.01, Table 2) reduced nitrogen concentration on average (Fig. 4A), which decreased further when the treatment was prolonged to 35 days (Fig. 4B). The reduction of total shoot nitrogen varied after 35 days of treatment and the interaction of treatments with both between-species and within-species reached significance. This reduction was greatest in the small-seeded pea NPE and lentil ATC with 35 days of waterlogging. With 21 days of recovery following waterlogging, the nitrogen level in both pea genotypes and grasspea had recovered to the level of plants grown continuously in drained soil (Fig. 4B). However, the lentil genotypes did not recover similarly.

## Discussion

The results demonstrated a significant variation in waterlogging tolerance within legumes. In contrast to other studies, in which seedlings of food legumes were exposed to waterlogging (Davies *et al*. 2000; Solaiman *et al*. 2007; Palta *et al*. 2010; Bramley *et al*. 2011), the current study focussed on transient waterlogging tolerance: after aerobic imbibition where seeds were germinated on the surface of waterlogged soil, and their growth in waterlogged soil and recovery from waterlogging stress were assessed.

### Variation in waterlogging tolerance among legume genotypes

Genetic variation in waterlogging tolerance within legume species has been demonstrated in lupin (Davies *et al*. 2000), faba bean (Solaiman *et al*. 2007), soya bean (Henshaw *et al*. 2007; Youn *et al*. 2008), lotus (Real *et al*. 2008), chickpea (Palta *et al*. 2010) and in the current study in both lentil and pea. The genotypes for this experiment were selected as contrasting pairs of pea and lentil with one small-seeded genotype each from South Asia, where there is a history of relay-sowing and germination under waterlogged conditions, and the other genotype—a larger-seeded Australian cultivar.

Following germination, important traits for waterlogging tolerance are the ability to increase root porosity during waterlogging, and for roots to recover during transient waterlogging; and to maintain shoot nitrogen and leaf chlorophyll (Malik *et al*. 2001). Considering these traits, the large-seeded genotypes from Australia (pea–Kaspa and lentil–Nugget) demonstrated greater waterlogging tolerance than their contrasting small-seeded pairs originating from South Asia (pea–NPE and lentil–ATC), presumably, due to greater carbohydrate pool and early vigour.

Enhanced root porosity contributes to better aeration in the root system in waterlogged soil (Colmer 2003). In both food (Solaiman *et al*. 2007) and pasture legumes (Gibberd *et al*. 1999) higher root porosity was observed in tolerant genotypes compared with sensitive genotypes. In the current experiment, tolerant legume cultivars showed enhanced root porosity by the formation of aerenchyma when grown in waterlogged substrate compared with sensitive genotypes. We confirmed the presence of aerenchyma by cross section of the main axis of the root (data not shown). Root porosity increased (up to 10 %) with growth in waterlogged conditions; however, constitutive root porosity was very low (∼2 %) and these values are much lower than those reported for waterlogging-tolerant legumes (Gibberd *et al*. 2001); all the species (and genotypes within species) responded to the treatment by increasing porosity with different degree of augmentation. Formation of adventitious roots is one trait that contributes to waterlogging tolerance; however, we did not observe adventitious root development in any of the legume genotypes; although, there is evidence for lentil to form adventitious roots when grown in waterlogged substrate (Erskine *et al*. 1994; Solaiman *et al*. 2007). In pea there are no reports of the development of adventitious roots (Jackson 1979). The discrepancy of the results from previous findings could be due to the experimental conditions. In the current study, the seeds were germinated aerobically on waterlogged soil in contrast to earlier experiments where 12- to 21-day-old seedlings were exposed to waterlogging.

Despite enhanced root porosity, waterlogging restricted root penetration into the substrate of pea and lentil (present study), wheat (Malik *et al*. 2001, 2002), soya bean (Henshaw *et al*. 2007), lupin (Davies *et al*. 2000; Real *et al*. 2008; Bramley *et al*. 2011) and chickpea (Palta *et al*. 2010). Restricted main root length was due to inadequate O_2_ diffusion to the root tip in waterlogged soil. According to the model of Armstrong (1979) with a given porosity (4–8 %, see Fig. 2) O_2_ could only diffuse down to 80–160 mm without any radial loss of oxygen along the root length. This demonstrates that the radial leakage of O_2_ along the root length and/or higher O_2_ uptake rate restricted roots to achieve desired root length. In contrast, grasspea main root length was not restricted by waterlogging (Fig. 1A). Presumably, grasspea formed a barrier to radial loss of O_2_. Solaiman *et al*. (2007) demonstrated the greater waterlogging tolerance of grasspea compared with lentil and pea. Further research is warranted to confirm the hypothesis.

Decreased shoot nitrogen in response to waterlogging has been well documented—wheat (Huang *et al*. 1994; Malik *et al*. 2001, 2002), soya bean (Riche 2004) and food legumes (Solaiman *et al*. 2007). Nitrogen reduction varied between legume genotypes under waterlogging due to effects on nitrogen fixation and the number of nodules (Cannell *et al*. 1979; Bacanamwo and Purcell 1999; Riche 2004; Bedard-Haughn 2009). The reduction of shoot nitrogen in waterlogged crops was due to a decrease in nutrient uptake (Malik *et al*. 2002; Solaiman *et al*. 2007; Palta *et al*. 2010). Waterlogging causes stelar anoxia to develop in the root (Aguilar *et al*. 2003), which reduced the loading of nutrients into the translocation stream, and restricted nitrogen supply to the shoot (Malik *et al*. 2001). In the current experiment, those genotypes able to enhance root porosity under waterlogging managed to maintain shoot N better—as in lotus (James and Sprent 1999; Striker *et al*. 2012, 2014) and soya bean (Shimamura *et al*. 2003; Thomas *et al*. 2005)—than those with low root porosity under waterlogging. Variation within species in chlorophyll retention in waterlogging conditions was reported in soya bean (Youn *et al*. 2008) and canola (Ashraf and Mehmood 1990), and tolerant genotypes with higher chlorophyll concentration identified (Talbot *et al*. 1987; Ashraf and Mehmood 1990; Pang *et al*. 2004; Youn *et al*. 2008). Once again, in this respect pea–Kaspa and lentil–Nugget showed better performance than other genotype pairs while the control grasspea also performed well.

### Effect of waterlogging on growth

Waterlogging reduced shoot growth in wheat (Malik *et al*. 2002), lotus (Mendoza *et al*. 2005) and chickpea (Palta *et al*. 2010). Other studies mentioned no significant effect of waterlogging on shoot growth in lupin, pea, lentil, faba bean, chickpea and grasspea during the treatment period (i.e. 7 days), but the effect of waterlogging appeared on plants during the recovery period (Yu and Rengel 1999; Solaiman *et al*. 2007). We did not find an effect of waterlogging on shoot and root mass in our experiment. In previous studies, seedlings of 12–21 days were exposed to waterlogging; however, in the current experiment seeds were germinated on waterlogged soil and exposed to soil waterlogging at initial establishment; and as the water table in the pots was 10 mm below the soil surface, roots accessed aerobic conditions on the top layer of the substrate. Increase in shoot dry weight in wheat has been reported when exposed to a short period (e.g. 8 days) of waterlogging due to carbohydrate accumulation (Trought and Drew 1980). Moreover, roots responded to waterlogging by altering their distribution pattern and producing numerous lateral roots (visual observation). These roots grew close to the soil surface to obtain oxygen under waterlogging conditions (Voesenek *et al*. 1999), as was found in various subspecies of maize (*Zea mays* ssp. *huehuetenangensis*) (Mano *et al*. 2005*a*, *b*) and in *Brassica napus* (Cannell and Belford 1980).

### Recovery from waterlogging

Root length recovers from waterlogging once allowed to grow in drained conditions. In wheat, stored carbohydrate was preferentially allocated to the re-growth of the root system during recovery (Malik *et al*. 2001, 2002). Root length recovered to the control value for tolerant legumes in the present experiment. This resulted in increasing net uptake of nitrogen transported to the shoot (Buwalda *et al*. 1988) and recovered leaf chlorophyll. Plants had to direct their energies into renewed pigment production, and re-greened chlorotic leaves at the onset of recovery (Smethurst *et al*. 2005). A previous study showed that pea and grasspea did not recover within 10 days after termination of waterlogging (Solaiman *et al*. 2007). Presumably, in our study, a longer recovery period led to the different result.

In the present study, relatively waterlogging-tolerant genotypes had an altered root distribution (i.e. near the soil surface) pattern while grown in waterlogged conditions as demonstrated by shallow root system-root length was short (∼100 mm) in waterlogged plants. But the overall root dry weight was similar for both drained and waterlogged treatments. However, there are disadvantages to the formation of the lateral roots—Armstrong *et al*. (1983) in pea and Malik *et al*. (unpubl. data) in wheat demonstrated that the lateral roots consumed O_2_ which restrict O_2_ movement through aerenchyma in the primary root; thus restrict root penetration into the deeper zone. However, in the current experiment plants maintained growth during the stress periods; presumably, the lateral roots become functional roots as demonstrated for pasture legumes (Gibberd *et al*. 1999). It is promising that during the recovery period the shallow root resumed growth and reached the same length as in the drained control, allowing access to soil moisture at depth as the soil profile dries later in the season.

## Conclusion

The present study with a limited number of legume genotypes identified variations in tolerance to transient waterlogging and its recovery between legume crops and also intra-species variation in pea and lentil—associated with seed mass. Waterlogging-tolerant legume genotypes had high root porosity, were relatively unaffected in shoot nitrogen content under waterlogging and in recovery could resume root growth and rapidly regain chlorophyll concentration to control levels. Clearly, there is substantial potential to select in a wider range of both pea and lentil germplasm for increased levels of waterlogging tolerance. Further investigation should first focus on evaluation of a large number of genotypes within each species of legumes to determine the genetic variation, followed by physiological assessment using contrasting genotypes.

## Sources of Funding

Our work was supported by the Centre for Plant Genetics and Breeding (PGB), University of Western Australia and project CIM-2009-038 funded by the Australian Centre for International Agriculture Research (ACIAR).

## Contributions by the Authors

T.I.A., A.I.M. and W.E. designed the research and analysed data; T.I.A. performed the experimentation in partial fulfilment of her MSc thesis project and A.I.M. drafted the manuscript. All Authors agreed to the final version of the manuscript.

## Conflict of Interest Statement

None declared.

## References

[PLV040C1] Aguilar EA Turner DW Gibbs DJ Armstrong W Sivasithamparam K 2003 Oxygen distribution and movement, respiration and nutrient loading in banana roots (*Musa* spp. L.) subjected to aerated and oxygen-depleted environments. Plant and Soil 253:91–102. 10.1023/A:1024598319404

[PLV040C3] Ali M Singh KK Pramanik SC Ali MO 2009 Cropping systems and production agronomy. In: Erskine W Muehlbauer FJ Sarker A Sharma B, eds. The lentil: botany, production and uses. Oxford, UK: CABI, 213–228.

[PLV040C2] Ali MO 2011 Enhancing lentil (*Lens culinaris* Medik.) production through relay cropping in transplant aman rice in medium low lands of Bangladesh. PhD Thesis, University of Rajshahi, Bangladesh.

[PLV040C4] Armstrong W 1979 Aeration in higher plants. Advances in Botanical Research 7:226–332.

[PLV040C5] Armstrong W Healy MT Lythe S 1983 Oxygen diffusion in pea II. Oxygen concentrations in the primary pea root apex as affected by growth, the production of laterals and radial oxygen loss. New Phytologist 94:549–559. 10.1111/j.1469-8137.1983.tb04864.x

[PLV040C6] Ashraf M Mehmood S 1990 Effects of waterlogging on growth and some physiological parameters of four Brassica species. Plant and Soil 121:203–209. 10.1007/BF00012313

[PLV040C7] Awadhwal N Gowda C Chauhan Y Flower D Haware M Rego T Pande S Saxena N Shanower T Johansen C 2001 Establishment of legumes following rice–a review. Natural Resource Management Program Report Vol. 2. pp 55.

[PLV040C8] Bacanamwo M Purcell LC 1999 Soybean dry matter and N accumulation responses to flooding stress, N sources and hypoxia. Journal of Experimental Botany 50:689–696. 10.1093/jxb/50.334.689

[PLV040C9] Bedard-Haughn A 2009 Managing excess water in Canadian prairie soils: a review. Canadian Journal of Soil Science 89:157–168. 10.4141/CJSS07071

[PLV040C10] Bramley H Tyerman SD Turner DW Turner NC 2011 Root growth of lupins is more sensitive to waterlogging than wheat. Functional Plant Biology 38:910–918. 10.1071/FP11148 32480948

[PLV040C11] Buwalda F Barrett-Lennard EG Greenway H Davies BA 1988 Effects of growing wheat in hypoxic nutrient solutions and of subsequent transfer to aerated solutions. II. Concentrations and uptake of nutrients and sodium in shoots and roots. Functional Plant Biology 15:599–612.

[PLV040C12] Cannell RQ Belford RK 1980 Effects of waterlogging at different stages of development on the growth and yield of winter oilseed rape (*Brassica napus* L.). Journal of the Science of Food and Agriculture 31:963–965. 10.1002/jsfa.2740310915

[PLV040C13] Cannell RQ Gales K Snaydon RW Suhail BA 1979 Effects of short-term waterlogging on the growth and yield of peas (*Pisum sativum*). Annals of Applied Biology 93:327–335. 10.1111/j.1744-7348.1979.tb06549.x

[PLV040C14] Colmer TD 2003 Long-distance transport of gases in plants: a perspective on internal aeration and radial oxygen loss from roots. Plant, Cell and Environment 26:17–36. 10.1046/j.1365-3040.2003.00846.x

[PLV040C15] Crawford RMM 1977 Tolerance of anoxia and ethanol metabolism in germinating seeds. New Phytologist 79:511–517. 10.1111/j.1469-8137.1977.tb02235.x

[PLV040C16] Davies CL Turner DW Dracup M 2000 Yellow lupin (*Lupinus luteus*) tolerates waterlogging better than narrow-leafed lupin (*L. angustifolius*) I. Shoot and root growth in a controlled environment. Australian Journal of Agricultural Research 51:701–709. 10.1071/AR99073

[PLV040C17] Erskine W 2009 Global production, supply and demand. In: Erskine W Muehlbauer FJ Sarker A Sharma B, eds. The lentil: botany, production and uses. Oxford, UK: CABI, 4–12.

[PLV040C18] Erskine W Tufail M Russell A Tyagi MC Rahman MM Saxena MC 1994 Current and future strategies in breeding lentil for resistance to biotic and abiotic stresses. Euphytica 73:127–135. 10.1007/BF00027189

[PLV040C19] Food and Agriculture Organization (FAO). 2013 Food and Agriculture Organisation of the United Nations. Rome, Italy http://faostat.fao.org.

[PLV040C20] Gibberd MR Colmer TD Cocks PS 1999 Root porosity and oxygen movement in waterlogging-tolerant *Trifolium tomentosum* and -intolerant *Trifolium glomeratum*. Plant, Cell and Environment 22:1161–1168. 10.1046/j.1365-3040.1999.00472.x

[PLV040C21] Gibberd M Gray JD Cocks PS Colmer TD 2001 Waterlogging tolerance among a diverse range of Trifolium accessions is related to root porosity, lateral root formation and ‘aerotropic rooting’. Annals of Botany 88:579–589. 10.1006/anbo.2001.1506

[PLV040C22] Henshaw TL Gilbert RA Scholberg JMS Sinclair TR 2007 Soya bean (*Glycine max* L. Merr.) genotype response to early-season flooding: I. Root and nodule development. Journal of Agronomy and Crop Science 193:177–188. 10.1111/j.1439-037X.2007.00257.x

[PLV040C23] Huang B Johnson JW Nesmith S Bridges DC 1994 Growth, physiological and anatomical responses of two wheat genotypes to waterlogging and nutrient supply. Journal of Experimental Botany 45:193–202. 10.1093/jxb/45.2.193

[PLV040C24] Jackson MB 1979 Rapid injury to peas by soil waterlogging. Journal of the Science of Food and Agriculture 30:143–152. 10.1002/jsfa.2740300208

[PLV040C25] Jackson MB Colmer TD 2005 Response and adaptation by plants to flooding stress. Annals of Botany 96:501–505. 10.1093/aob/mci205 16217870 PMC4247020

[PLV040C26] James EK Sprent JI 1999 Development of N_2_-fixing nodules on the wetland legume *Lotus uliginosus* exposed to conditions of flooding. New Phytologist 142:219–231. 10.1046/j.1469-8137.1999.00394.x

[PLV040C27] Malik AI Colmer TD Lambers H Schortemeyer M 2001 Changes in physiological and morphological traits of roots and shoots of wheat in response to different depths of waterlogging. Functional Plant Biology 28:1121–1131. 10.1071/PP01089

[PLV040C28] Malik AI Colmer TD Lambers H Setter TL Schortemeyer M 2002 Short-term waterlogging has long-term effects on the growth and physiology of wheat. New Phytologist 153:225–236. 10.1046/j.0028-646X.2001.00318.x

[PLV040C29] Malik AI Ali MO Zaman MS Flower K Rahman MM Erskine W 2015 Relay sowing of lentil (*Lens culinaris* subsp. *culinaris*) to intensify rice-based cropping. The Journal of Agricultural Science 10.1017/S0021859614001324.

[PLV040C30] Mano Y Muraki M Fujimori M Takamizo T Kindiger B 2005a Identification of QTL controlling adventitious root formation during flooding conditions in teosinte (*Zea mays* ssp. *huehuetenangensis*) seedlings. Euphytica 142:33–42. 10.1007/s10681-005-0449-2

[PLV040C31] Mano Y Omori F Muraki M Takamizo T 2005b QTL mapping of adventitious root formation under flooding conditions in tropical maize (*Zea mays* L.) seedlings. Breeding Science 55:343–347. 10.1270/jsbbs.55.343

[PLV040C32] Mendoza R Escudero V García I 2005 Plant growth, nutrient acquisition and mycorrhizal symbioses of a waterlogging tolerant legume (*Lotus glaber* Mill.) in a saline-sodic soil. Plant and Soil 275:305–315. 10.1007/s11104-005-2501-3

[PLV040C33] Palta JA Ganjeali A Turner NC Siddique KHM 2010 Effects of transient subsurface waterlogging on root growth, plant biomass and yield of chickpea. Agricultural Water Management 97:1469–1476. 10.1016/j.agwat.2010.05.001

[PLV040C34] Pang J Zhou M Mendham N Shabala S 2004 Growth and physiological responses of six barley genotypes to waterlogging and subsequent recovery. Australian Journal of Agricultural Research 55:895–906. 10.1071/AR03097

[PLV040C35] Patrick W Gambrell R Faulkner S Sparks D Page A Helmke P Loeppert R Soltanpour P Tabatabai M Johnston C 1996 Redox measurements of soils. In: Sparks DL Page AL Helmke PA Loeppert RH, eds. Methods of soil analysis. Part 3—chemical methods. Madison, WI: Soil Science Society of America, American Society of Agronomy, 1255–1273.

[PLV040C36] Purseglove JW 1968 Tropical crops dicotyledons. London, UK: Longman.

[PLV040C37] Ramakrishna A Gowda CLL Johansen C 2000 Management factors affecting legumes production in the Indo-Gangetic Plain. In: Legumes in rice and wheat cropping systems of the Indo-Gangetic Plain-constraints and opportunities. Patancheru, Andhra Pradesh: ICRISAT, 156–165.

[PLV040C38] Raskin I 1983 A method for measuring leaf volume, density, thickness, and internal gas volume. HortScience 18:698–699.

[PLV040C39] Real D Warden J Sandral GA Colmer TD 2008 Waterlogging tolerance and recovery of 10 Lotus species. Australian Journal of Experimental Agriculture 48:480–487. 10.1071/EA07110

[PLV040C40] Riche CJ 2004 Identification of soybean cultivars tolerance to waterlogging through analyses of leaf nitrogen concentration. MSc Thesis Louisiana State University.

[PLV040C41] Sarlistyaningsih L Sivasithamparam K Setter TL 1995 Influence of waterlogging on germination and survival of lupin seeds (*Lupinus angustifolius* L. cv. Gungurru) coated with calcium peroxide and streptomycin. Australian Journal of Experimental Agriculture 35:537–541. 10.1071/EA9950537

[PLV040C42] Setter TL Waters I 2003 Review of prospects for germplasm improvement for waterlogging tolerance in wheat, barley and oats. Plant and Soil 253:1–34. 10.1023/A:1024573305997

[PLV040C43] Shimamura S Mochizuki T Nada Y Fukuyama M 2003 Formation and function of secondary aerenchyma in hypocotyl, roots and nodules of soybean (*Glycine max*) under flooded conditions. Plant and Soil 251:351–359. 10.1023/A:1023036720537

[PLV040C44] Siddique KHM Sykes J 1997 Pulse production in Australia past, present and future. Australian Journal of Experimental Agriculture 37:103–111. 10.1071/EA96068

[PLV040C45] Siddique KHM Walton GH Seymour M 1993 A comparison of seed yields of winter grain legumes in Western Australia. Australian Journal of Experimental Agriculture 33:915–922. 10.1071/EA9930915

[PLV040C46] Smethurst CF Garnett T Shabala S 2005 Nutritional and chlorophyll fluorescence responses of lucerne (*Medicago sativa*) to waterlogging and subsequent recovery. Plant and Soil 270:31–45. 10.1007/s11104-004-1082-x

[PLV040C47] Solaiman Z Colmer TD Loss SP Thomson BD Siddique KHM 2007 Growth responses of cool-season grain legumes to transient waterlogging. Australian Journal of Agricultural Research 58:406–412. 10.1071/AR06330

[PLV040C48] Striker GG Izaguirre RF Manzur ME Grimoldi AA 2012 Different strategies of *Lotus japonicus*, *L. corniculatus* and *L. tenuis* to deal with complete submergence at seedling stage. Plant Biology 14:50–55. 21972978 10.1111/j.1438-8677.2011.00493.x

[PLV040C49] Striker GG Casas C Manzur ME Ploschuk RA Casal JJ 2014 Phenomic networks reveal largely independent root and shoot adjustment in waterlogged plants of *Lotus japonicus*. Plant, Cell and Environment 37:2278–2293. 10.1111/pce.12268 24393069

[PLV040C50] Talbot RJ Etherington JR Bryant JA 1987 Comparative studies of plant growth and distribution in relation to waterlogging. XII. Growth, photosynthetic capacity and metal ion uptake in *Salix caprea* and *S. cinerea* ssp. *Oleifolia*. New Phytologist 105:563–574. 10.1111/j.1469-8137.1987.tb00894.x

[PLV040C51] Thomas AL Guerreiro SMC Sodek L 2005 Aerenchyma formation and recovery from hypoxia of the flooded root system of nodulated soybean. Annals of Botany 96:1191–1198. 10.1093/aob/mci272 16199486 PMC4247071

[PLV040C52] Thomson CJ Armstrong W Waters I Greenway H 1990 Aerenchyma formation and associated oxygen movement in seminal and nodal roots of wheat. Plant, Cell and Environment 13:395–403. 10.1111/j.1365-3040.1990.tb02144.x

[PLV040C53] Trought MCT Drew MC 1980 The development of waterlogging damage in young wheat plants in anaerobic solution cultures. Journal of Experimental Botany 31:1573–1585. 10.1093/jxb/31.6.1573

[PLV040C54] Voesenek LACJ Armstrong W Colmer TD Bögemann GM McDonald MP 1999 A lack of aerenchyma and high rates of radial oxygen loss from the root base contribute to the waterlogging intolerance of *Brassica napus*. Functional Plant Biolology 26:87–93.

[PLV040C55] White P Seymour M Burgess P Harries M 2005 Producing pulses in the southern agricultural region. South Perth, WA: Department of Agriculture, Western Australia; and Grains Research & Development Corporation, pp 132.

[PLV040C56] Youn JT Van K Lee JE Kim WH Yun HT Kwon YU Ryu YH Lee SH 2008 Waterlogging effects on nitrogen accumulation and N_2_ fixation of supernodulating soybean mutants. Journal of Crop Science and Biotechnology 11:111–118.

[PLV040C57] Yu Q Rengel Z 1999 Waterlogging influences plant growth and activities of superoxide dismutases in narrow-leafed lupin and transgenic tobacco plants. Journal of Plant Physiology 155:431–438. 10.1016/S0176-1617(99)80127-8

